# Supplementary material for: Genetic Potential of Newly Developed Maize Hybrids under Different Water-Availability Conditions in an Arid Environment
Source: Life (Basel). 2024 Mar 29;14(4):453. doi: 10.3390/life14040453 (PMC11051332; doi:10.3390/life14040453)
Supplement: Supplementary file 1 [file life-14-00453-s001.zip › life-2886997-supplementary.pdf]

Table S1. Mechanical and some chemical properties of the investigated soil sample.

Soil properly	Season 2019	Season 2020
Mechanical analysis:		
Coarse sand %	5.11	5.20
Fine sand %	25.18	23.47
Silt %	20.00	20.10
Clay %	49.71	51.23
Textural class	Clay	Clay
Chemical analysis:		
pH(1:2.5)	7.95	7.92
Organic matter g/kg^{-1}	17.1	19.3
EC (dSm^{-1})	1.89	1.83
Total N%	0.15	0.18
Total P%	1.32	1.41
Total K%	0.50	0.53

Table S2. The applied drought tolerance indices

Drought tolerance indices	Equation	Reference
1- Mean productivity (MP)	MP= $\frac{(Yp+Ys)}{2}$	Rosielle and Hambling (1981)
2- Stress tolerance index (STI)	STI= $\frac{(Ys)(Yp)}{(\bar{Y}p)^2}$	Fernandez (1992)
3- Tolerance index (TOL)	TOL= Yp- Ys	Rosielle and Hambling (1981)
4- Stress sensitivity index (SSI)	SSI = $\frac{1-\left(\frac{Ys}{Yp}\right)}{1-\left((\frac{\bar{Y}s}{\bar{Y}b}\right)}$	Fischer and Maurer (1978)
5- Yield index (YI)	YI = $\frac{Y_s}{\bar{Y}s}$	Gavuzzi et al. (1977)
6- Yield stability index (YSI)	YSI = $\frac{Ys}{Yp}$	Bouslama and Schapaugh (1984)
7- Harmonic mean (HM)	HM = $\frac{2(Yp.Ys)}{Yp+Ys}$	Jafari et al. (2009)

Yp, Ys, $\bar{Y}p$, and $\bar{Y}s$ refers to yield under normal conditions, yield under stress, mean yield under normal irrigation, and mean yield under drought conditions, respectively.

Table S3. Mean performance of parents, crosses, and check hybrids for grain yield, number of rows per ear, number of grains per row, and 100 kernel weight under well-watered and stressed conditions.

Hybrid	Grain yield (t/ha)		Number of Rows/ear		Number of Grains/row		100 kernel weight (g)	
	W--Watered	D--Stress	W--Watered	D--Stress	W--Watered	D--Stress	W--Watered	D--Stress
L-1×T-1	9.018	7.564	13.87	11.83	32.87	22.67	29.67	29.33
L-2×T-1	10.66	10.41	13.73	13.33	37.37	36.33	29.33	28.67
L-4×T-1	8.825	8.763	13.07	13.00	36.67	31.33	27.33	25.00
L-5×T-1	11.25	11.20	11.00	11.67	37.43	33.33	31.67	29.00
L-6×T-1	12.07	11.23	13.33	12.87	37.00	35.73	26.67	24.00
L-7×T-1	13.52	12.49	15.00	14.50	41.07	34.67	37.33	37.33
L-8×T-1	10.81	10.72	12.07	12.47	36.27	34.00	34.67	32.00
L-9×T-1	11.42	9.711	12.67	11.93	35.53	35.00	31.67	29.00
L-10×T-1	9.235	8.190	12.67	10.33	37.60	34.33	33.00	33.67
L-11×T-1	12.79	12.49	15.33	14.67	40.27	38.20	36.00	35.33
L-1×T-2	13.88	12.76	15.47	15.87	37.73	37.47	40.33	35.00
L-2×T-2	10.09	8.852	13.67	14.00	23.33	22.33	35.00	30.33
L-4×T-2	7.526	7.433	14.33	16.00	13.33	12.00	35.67	27.33
L-5×T-2	9.417	8.634	14.73	13.67	28.27	27.67	35.67	30.33
L-6×T-2	10.62	10.59	13.47	14.00	33.93	33.33	36.00	29.00
L-7×T-2	10.07	10.00	13.67	13.27	28.67	27.60	33.67	31.00
L-8×T-2	10.32	8.511	13.80	13.53	32.80	32.53	33.67	31.67
L-9×T-2	10.00	9.060	12.77	13.50	34.93	34.57	30.67	30.00
L-10×T-2	9.792	8.574	12.53	11.93	39.13	37.73	32.67	32.67
L-11×T-2	12.66	12.62	14.33	16.33	41.60	41.33	39.33	38.20
Parental lines								
L-1	4.345	2.864	14.00	14.00	13.67	12.33	28.33	30.33
L-2	4.703	3.081	11.07	11.67	13.00	13.00	30.00	29.67
L-4	4.574	2.803	13.67	12.33	13.00	11.67	28.67	28.33
L-5	2.882	2.782	12.33	12.33	11.67	10.33	28.33	23.33
L-6	3.661	2.935	13.00	12.00	21.33	19.33	24.67	24.00
L-7	5.148	3.501	12.33	13.33	21.33	19.67	27.00	27.00
L-8	3.162	2.636	12.00	10.67	10.00	9.67	30.00	29.33
L-9	3.507	3.835	10.33	9.00	17.80	19.43	28.00	26.00
L-10	3.850	3.562	11.67	11.00	22.67	21.17	31.33	29.67
L-11	3.335	3.206	12.00	11.43	16.67	17.77	30.33	25.00
T-1	2.881	2.518	9.33	9.00	9.33	9.00	28.33	28.33
T-2	3.628	3.225	10.67	11.73	11.50	11.00	27.33	28.33
Check hybrids								
SC 10	11.23	10.45	13.67	13.33	34.67	33.00	33.33	34.00
SC 30K8	11.42	10.70	13.50	13.40	35.00	33.73	35.00	34.67
LSD 0.05	0.961	1.052	1.69	1.44	3.53	4.13	4.27	3.60
LSD 0.01	1.260	1.380	2.21	1.89	4.62	5.42	5.59	4.73

Table S4. Mean performance of parents, crosses, and check varieties for plant height, ear height, days to tasseling, days to silking, and chlorophyll content under well-watered and stressed conditions.

Hybrid	Plant height (cm)		Ear height (cm)		Days to tasseling		Days to silking		Chlorophyll content (SPAD value)	
	W--Watered	D--Stress	W--Watered	D--Stress	W--Watered	D--Stress	W--Watered	D--Stress	W--Watered	D--Stress
L-1×T-1	206.0	204.0	88.00	86.67	61.00	61.33	62.67	63.67	49.73	41.33
L-2×T-1	237.3	219.3	105.33	104.7	56.33	60.67	60.67	63.33	44.67	44.13
L-4×T-1	253.7	251.0	128.67	126.3	56.00	56.33	60.67	62.33	48.17	42.17
L-5×T-1	256.0	253.3	122.33	121.3	57.67	59.33	62.67	63.67	47.10	43.33
L-6×T-1	250.0	246.7	135.00	132.7	54.67	56.33	59.67	62.00	48.30	46.47
L-7×T-1	252.7	248.3	128.33	121.7	56.00	56.67	59.67	61.70	57.17	50.67
L-8×T-1	256.7	251.7	130.67	113.3	55.00	62.67	60.00	63.00	47.97	46.97
L-9×T-1	252.0	246.3	128.67	126.7	55.67	59.00	61.67	64.00	42.33	41.67
L-10×T-1	231.3	228.3	112.00	108.0	50.67	51.67	58.33	60.67	42.77	36.67
L-11×T-1	248.0	241.7	127.33	122.0	56.67	57.33	60.67	61.67	51.33	49.60
L-1×T-2	262.0	250.7	142.67	123.3	57.33	60.33	62.67	63.67	61.67	56.77
L-2×T-2	248.3	241.7	105.00	96.67	60.00	66.00	67.00	71.67	50.00	47.77
L-4×T-2	195.3	193.7	103.33	95.33	57.00	58.67	64.33	67.33	44.33	43.67
L-5×T-2	231.7	226.7	108.67	106.0	57.67	58.67	61.67	65.33	46.67	43.33
L-6×T-2	284.3	280.0	144.00	142.7	54.67	55.00	59.33	62.33	55.00	52.53
L-7×T-2	256.7	253.3	138.00	136.7	57.67	59.67	63.67	66.00	45.80	43.33
L-8×T-2	254.0	248.0	142.67	142.3	56.33	60.67	65.00	64.67	46.43	45.03
L-9×T-2	282.0	276.7	139.33	129.0	60.00	63.33	66.33	71.00	47.87	45.30
L-10×T-2	251.3	246.0	139.33	128.7	55.00	56.00	59.00	62.33	47.33	42.00
L-11×T-2	263.3	260.7	133.33	132.7	57.33	59.67	61.67	65.00	50.13	58.67
Parental lines										
L-1	141.7	141.3	84.67	83.00	61.00	62.67	65.00	67.00	43.27	39.40
L-2	177.0	176.7	81.33	81.00	60.00	61.67	62.67	65.33	41.63	41.13
L-4	146.7	145.0	86.67	77.67	59.33	60.67	64.33	65.33	42.33	38.10
L-5	161.7	161.0	96.67	90.33	61.67	63.00	65.67	67.33	42.67	39.00
L-6	173.3	173.3	97.00	90.67	63.00	63.33	67.67	69.00	45.50	44.20
L-7	178.7	175.0	87.00	81.00	64.67	65.67	67.33	68.33	50.10	47.60
L-8	150.0	148.3	85.33	84.33	63.33	66.00	70.00	70.33	41.80	33.07
L-9	197.3	194.0	85.33	83.33	62.33	63.00	68.67	69.33	43.27	42.03
L-10	146.3	145.7	88.33	85.33	55.33	63.00	65.33	70.33	39.87	38.80
L-11	179.0	176.7	86.00	75.33	56.67	64.33	65.33	67.67	45.10	44.50
T-1	156.0	153.3	87.00	85.67	60.67	61.67	67.00	68.00	41.67	40.67
T-2	156.0	152.7	89.33	80.33	63.00	63.67	67.67	68.33	43.33	42.77
Check hybrids										
SC 10	260.7	254.0	139.33	131.0	58.67	60.00	63.67	65.00	49.27	45.27
SC 30K8	225.0	224.7	115.00	113.3	56.33	59.00	61.67	64.00	50.67	47.50
LSD 0.05	15.64	12.34	9.95	10.30	3.56	3.10	2.80	3.03	5.67	4.93
LSD 0.01	20.51	16.18	13.04	13.50	4.67	4.06	3.67	3.98	7.43	6.47

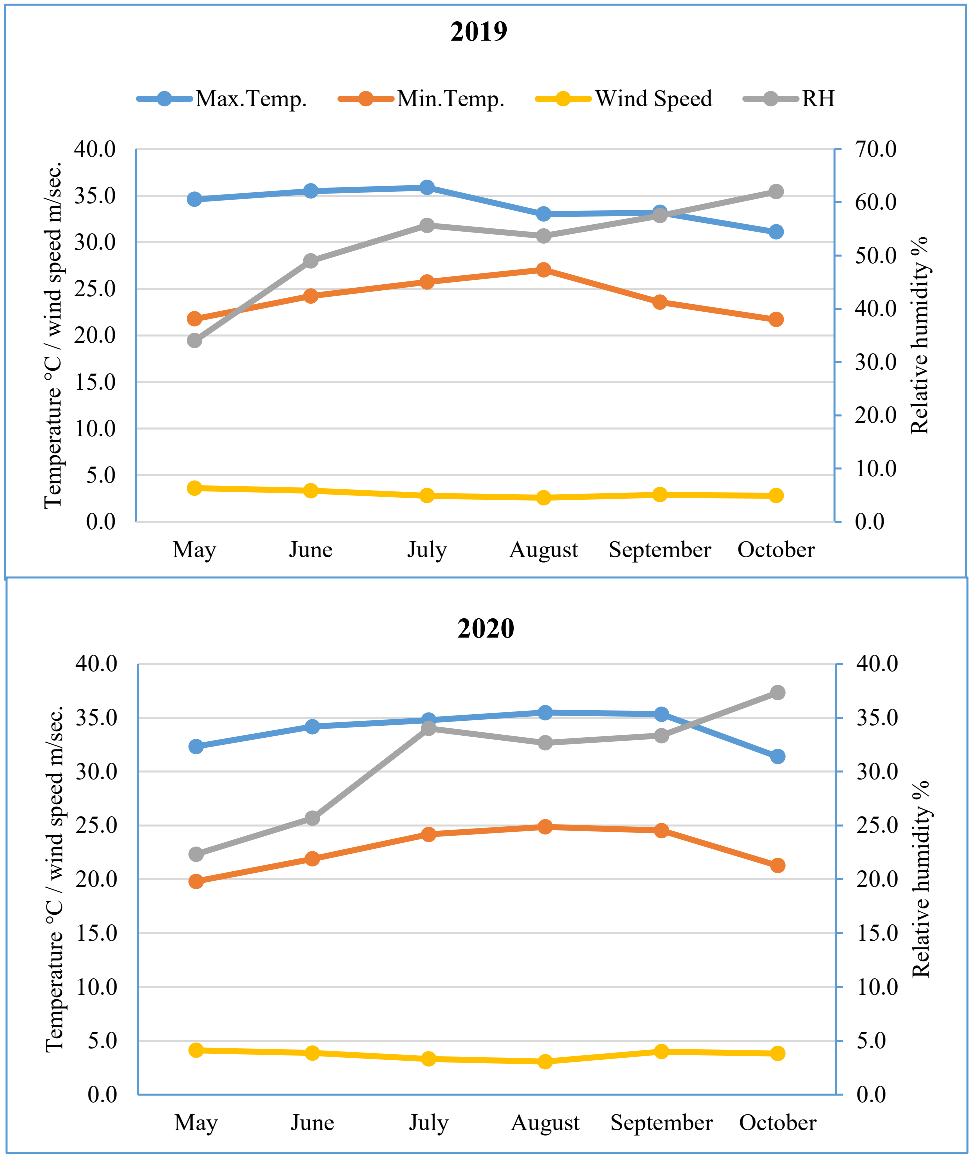

Figure S1. Monthly air temperature, relative humidity, and wind speed in two seasons
